# Microvascular Disease in the Hippocampus in Obstructive Sleep Apnoea

**DOI:** 10.3390/clockssleep8030051

**Published:** 2026-08-27

**Authors:** Cuicui Xu, Jessica E. Owen, Thorarinn Gislason, Bryndís Benediktsdóttir, Jiming Ye, Stephen R. Robinson

**Affiliations:** 1 School of Health and Biomedical Sciences, Royal Melbourne Institute of Technology (RMIT) University, Bundoora, VIC 3083, Australia; cuicui.xu@wimr.org.au (C.X.); jessica.owen811@gmail.com (J.E.O.); jiming.ye@hotmail.com (J.Y.); 2 Department of Sleep, Landspitali—The National University Hospital of Iceland, 101 Reykjavik, Iceland; thorarig@landspitali.is (T.G.); brynben@hi.is (B.B.); 3 Medical Faculty, University of Iceland, 101 Reykjavik, Iceland; 4 Institute for Breathing and Sleep, Austin Health, Heidelberg, VIC 3084, Australia

**Keywords:** ageing, blood flow, capillary, CPAP, ischemia, intermittent hypoxia, oxidative stress

## Abstract

Ischemia is a primary cause of microvascular disease, particularly in the hippocampus, which is unusually vulnerable to hypoperfusion and hypoxia. Since intermittent hypoxia is a defining feature of obstructive sleep apnoea (OSA), the present study examined microvessels in autopsy tissue from patients with clinically confirmed OSA. Immunohistochemistry was performed on 30 hippocampal autopsy samples to investigate whether the incidence of microvascular abnormalities is associated with increased OSA severity, and if so, whether regular use of continuous positive airway pressure (CPAP) is protective. There were significantly more string vessels (4.3 ± 0.9 vs. 1.7 ± 0.4, *p* = 0.014), narrowing vessels (3.5 ± 1.0 vs. 0.9 ± 0.3, *p* = 0.024), strictures (2.6 ± 0.4 vs. 1.1 ± 0.2, *p* = 0.002) and total abnormalities (10.3 ± 1.5 vs. 3.7 ± 0.6, *p* = 0.001) in the CA1 subregion of the moderate–severe OSA group (mean ODI 35.9 ± 6.4 events/h sleep) than in the mild–moderate OSA group (mean ODI 18.5 ± 3.7 events/h sleep). There were no significant differences between regular CPAP users and non-CPAP users, or between younger and older individuals. We conclude that in moderate–severe OSA, the preponderance of microvascular abnormalities in the CA1, which are too narrow to carry blood, amplify its vulnerability to ischemia.

## 1. Introduction

OSA is characterized by chronic intermittent hypoxia [1], which is a major contributing factor to the pathogenesis of OSA-related comorbidities, such as systemic arterial hypertension, ischemic heart disease, and cardiac arrhythmias, all of which are associated with vascular dysfunction [2]. The chronic cycles of desaturation–reoxygenation induce oxidative stress [2], stimulate chronic inflammation [3], and increase the activity of the sympathetic nervous system due to the stress associated with hypoxic episodes [4]. These stressors have the potential to injure microvessels and trigger pathological changes in their morphology.

Shear stress, the friction created on vascular endothelial cells by blood flow, is critical for the survival and continued patency of vessels [5]. Shear stress diminishes when a vessel is partially occluded or if the upstream blood flow is slowed (hypoperfusion) [5]. String vessels are the remnants of capillaries that have collapsed and atrophied due to insufficient shear stress, leaving behind the basement membrane connective tissue, mainly composed of collagen IV [6]. These thin, non-functional strands of connective tissue mark the locations of dead capillaries. Because they lack endothelial cells and an open inner channel (lumen), string vessels do not carry blood. The increased numbers of string vessels in Alzheimer’s disease (AD) [6,7] may be related to the decreased cerebral blood flow that is characteristic of that disease [8,9,10]. Like in AD, neuroimaging studies of moderate–severe OSA patients have reported decreased rates of blood flow in the hippocampus and parahippocampal gyrus when compared to healthy controls [11,12,13]. This reduced blood flow raises the possibility that the numbers of string vessels may increase in the hippocampus as the severity of OSA increases.

In ultrastructural studies of cortical tissue from AD patients, terminal arterioles and capillaries display irregular shapes and have a series of focal constrictions and dilations, resulting in altered vessel diameter along their course [14,15,16]. Cerebral arterioles in white matter often become tortuous with ageing, and begin to feature coils, loops and spirals, which slow blood flow even if the lumen diameter remains constant [17]. Tortuous vessels are rare in the brains of preterm babies, children and young adults, and they start to appear after middle age [18]. The vessels become elongated due to an age-related decrease in elasticity, and since the end points of the vessels are fixed, the extra length becomes curved and twisted, forming tortuous vessels [19]. Moreover, the deposition of excessive collagen in vessel walls can occlude or narrow the lumina, further slowing blood flow [20]. Therefore, the presence of abnormal microvessels of any kind is indicative of ageing or disease, and in turn contributes to a slowing of blood flow and a consequent decline in neuronal function.

An autopsy study of OSA patients found that the more time a patient spent with a blood oxygen saturation below 95%, the higher the risk of microinfarcts in the brain [21]. Although microinfarcts are a consequence of cerebrovascular disease, that study did not investigate any specific characteristics of the microvasculature. An animal model of chronic IH demonstrated significant cerebrovascular dysfunction and oxidative stress, suggesting that these two factors could account for the increased risk of cerebrovascular disease in OSA patients [22]. Recently, our team [23] used quantitative immunohistochemistry to investigate whether microvascular remodelling occurs in the human hippocampus in response to OSA severity, patient age or CPAP use. We found little evidence of microvascular remodelling and concluded that the limited adaptability of the microvasculature may increase its vulnerability to hypoxic injury and microvascular disease. However, that study did not investigate whether hippocampal microvessels exhibit signs of ischemic injury, such as increased numbers of string vessels or tortuous vessels. Therefore, the current study aimed to investigate whether the incidence of diseased microvessels in the human hippocampus increases in association with OSA severity. Additionally, this study investigated whether the incidence of microvascular abnormalities is lower in regular CPAP users, and whether patient age is a contributing factor.

## 2. Results

**Figure 1:**
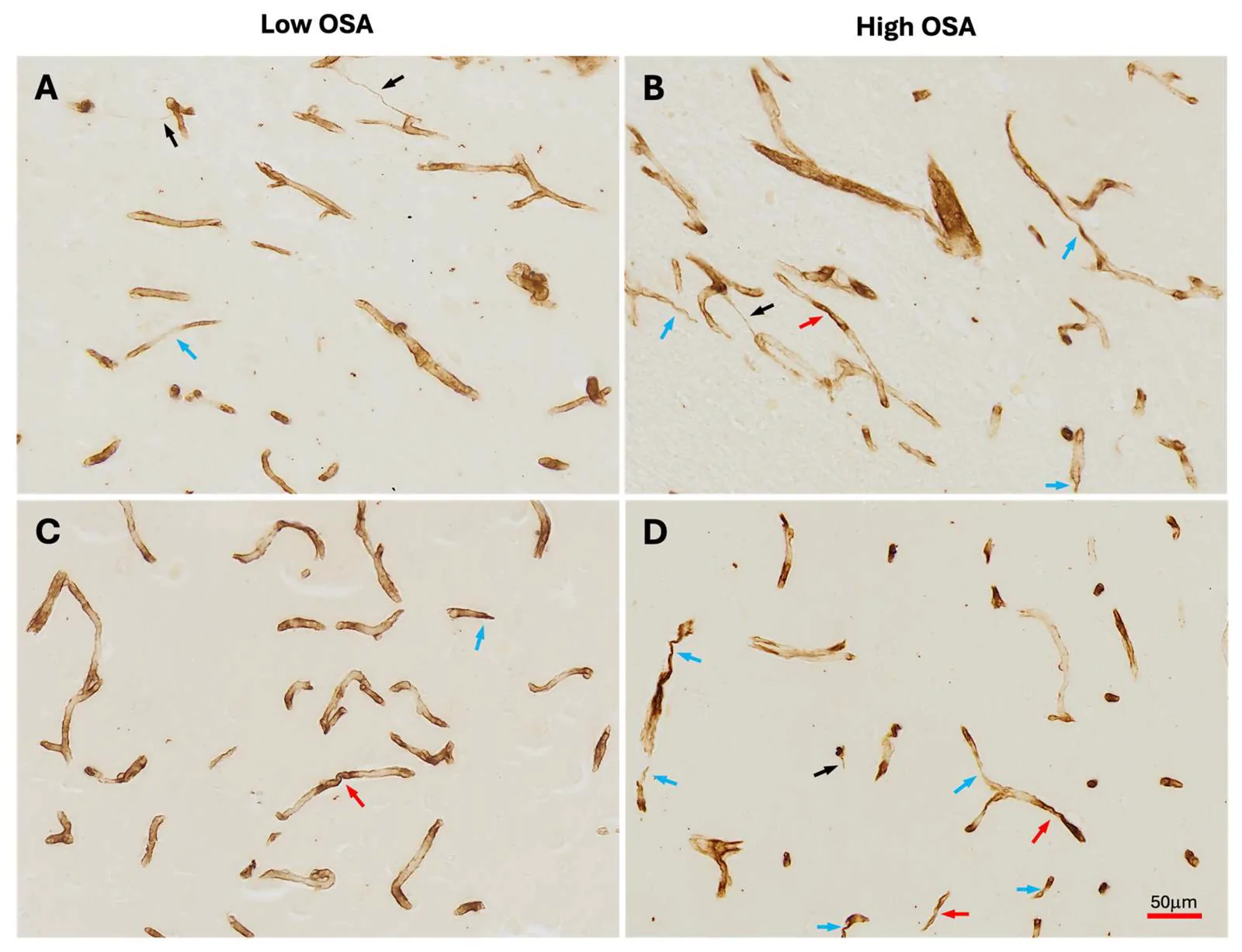
Examples of vessel profiles in low OSA ((**A**,**C**); ODI < 20 events/h sleep) and high OSA ((**B**,**D**); ODI ≥ 20 events/h sleep). Arrows indicate selected examples of abnormal vessels. Black arrows = string vessels; blue arrows = narrowing vessels; red arrows = strictures.

The study sample consisted of 30 of the 31 OSA patients described previously [23]; one sample was excluded because the tissue was refractory to double-immunostaining for Collagen IV and GluT-1. Figure 1 shows micrographs of tissues that have been double-immunostained for Collagen IV and GluT-1 in the CA1 in the low (ODI < 20 events/h sleep) and high (ODI ≥ 20 events/h sleep) OSA groups.

### 2.1. Associations Between OSA Severity and Microvascular Abnormalities

#### 2.1.1. Increased Incidence of Microvessel Abnormalities in the High OSA Group

A total of 3513 vessel segments were automatically selected by Automated Retinal Image Analyzer (ARIA, V1-09-12-11), in five hippocampal subregions of 30 OSA patients; of these, 1658 were found in the low OSA group and 1891 in the high OSA group. There were fewer microvessels in the fimbria than in the other four regions (Table 1).

**Figure 2:**
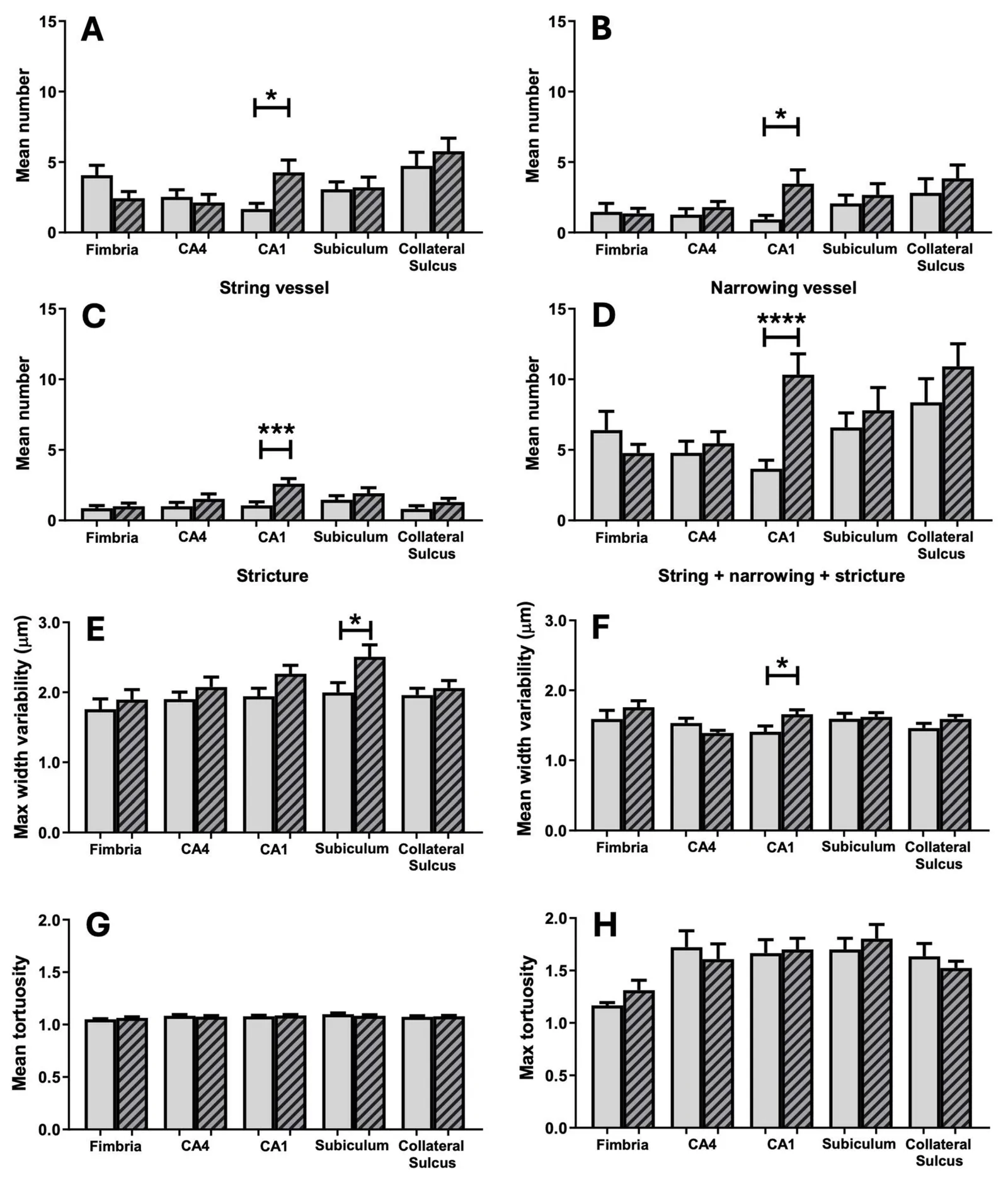
Associations between OSA severity and the frequency of abnormal microvessels in hippocampal subregions. (**A**) String vessel counts in low and high OSA groups across five hippocampal regions. (**B**) Narrowing vessel counts in low and high OSA groups across five hippocampal regions. (**C**) Stricture counts in low and high OSA groups across five hippocampal regions. (**D**) Total diseased vessel counts in low and high OSA groups across five hippocampal regions. The width variability measures (**E**,**F**) are indicators of dilated/constricted vessels, while the tortuosity measures (**G**,**H**) are indicators of tortuous vessels. Plain bars = low OSA (ODI < 20 events/h sleep); Striped bars = high OSA (ODI ≥ 20 events/h sleep). *p* ≤ 0.05 *; *p* ≤ 0.005 ***; *p* ≤ 0.001 ****.

The numbers of abnormal microvessels in the two groups in the five subregions are shown in Figure 2. In the CA1 the high OSA group had 2.56-fold more string vessels (4.3 ± 0.9 vs. 1.7 ± 0.4, 95% CI [0.59, 4.61], *t* (19.6) = 2.703, *p* = 0.014, unadjusted; Figure 2A) than the low OSA group. With 15 participants per group, a two-sided α = 0.05, and an estimated power of 80%, the estimated minimum detectable effect size (MDE; a measure of the study’s sensitivity at the given sample size) was *d* = 1.06 and the observed effect size was *d* = 0.99. The difference between groups is consistent with the conclusion that there are more string vessels in the CA1 of the high OSA group than in the low OSA group.

There were 3.71-fold more narrowing vessels in the high OSA group (3.5 ± 1.0 vs. 0.9 ± 0.3, 95% CI [0.37, 4.69], *t* (16.3) = 2.483, *p* = 0.024, unadjusted; Figure 2B) than in the low OSA group. With 15 participants per group, a two-sided α = 0.05, and an estimated power of 80%, the estimated MDE was *d* = 1.06 and the observed effect size was *d* = 0.9. The difference between groups is consistent with the conclusion that the high OSA group had more narrowing vessels in the CA1 than the low OSA group.

There were 2.44-fold more strictures in the high OSA group in the CA1 (2.6 ± 0.4 vs. 1.1 ± 0.2, 95% CI [0.63, 2.43], *t* (28) = 3.490, *p* = 0.002, unadjusted; Figure 2C). With 15 participants per group, a two-sided α = 0.05, and an estimated power of 80%, the estimated MDE was *d* = 1.06 and the observed effect size was *d* = 1.27. The difference between groups is consistent with the conclusion that the high OSA group had more strictures in the CA1 than the low OSA group.

As all three types of abnormal vessels were measured in the same way, and as each type will have little or no blood flow due to their constricted lumina, the total number of microvascular abnormalities in a standard field of view was measured by combining all three types of abnormal microvessels. In the CA1, there were 2.82-fold more string + narrowing + strictures (10.3 ± 1.5 vs. 3.7 ± 0.6, 95% CI [3.34, 9.99], *t* (18.5) = 4.202, *p* = 0.001, unadjusted; Figure 2D) in the high OSA group than in the low OSA group. With 15 participants per group, a two-sided α = 0.05, and an estimated power of 80%, the estimated MDE was *d* = 1.06 and the observed effect size was *d* = 1.53. The difference between groups is consistent with the conclusion that the high OSA group had more abnormal vessels in the CA1 than the low OSA group.

The proportion (%) of total microvessels that were abnormal varied between regions, as shown in Table 2, with the highest proportion being observed in the fimbria. In general, string vessels were the most common microvascular abnormality, while strictures were the least common.

#### 2.1.2. Increased Vessel Width Variability in the High-OSA Group

Since variability in capillary width along the course of a vessel can be an indicator of disease [14,15], the vessel width variability was compared between the low and high OSA groups. The maximum width variability (max. SD; µm) in the high OSA group was larger (1.17–1.26-fold) than in the low OSA group in the CA1 region (2.3 ± 0.1 vs. 1.9 ± 0.1, 95% CI [−0.02, 0.66], *t* (28) = 1.954, *p* = 0.061, unadjusted; Figure 2E). With 15 participants per group, a two-sided α of 0.05, and an estimated power of 80%, the estimated MDE was *d* = 1.06 and the observed effect size was *d* = 0.71. As the difference between groups was not significant, the null hypothesis, that OSA severity is not associated with greater maximum vessel width variability in the CA1, was retained.

A difference was observed in the subiculum (2.5 ± 0.2 vs. 2.0 ± 0.1, 95% CI [0.06, 0.96], *t* (28) = 2.334, *p* = 0.027, unadjusted; Figure 2E). With 15 participants per group, a two-sided α = 0.05, and an estimated power of 80%, the estimated MDE was *d* = 1.06 and the observed effect size was *d* = 0.85. The difference between groups is consistent with the conclusion that maximum vessel width variability in the subiculum is greater in the high OSA group.

The mean width variability (mean 2SD; µm) in the high OSA group was 1.18-fold larger than in the low OSA group in the CA1 region (1.7 ± 0.1 vs. 1.4 ± 0.1, 95% CI [0.04, 0.46], *t* (28) = 2.456, *p* = 0.021, unadjusted; Figure 2F). With 15 participants per group, a two-sided α = 0.05, and an estimated power of 80%, the estimated MDE was *d* = 1.06 and the observed effect size was *d* = 0.90. The difference between groups is consistent with the conclusion that the mean vessel width variability in the CA1 is greater in the high OSA group.

#### 2.1.3. Equal Tortuosity Between Low and High OSA Groups

Studies have reported that cerebral arterioles in older brains and some neurodegenerative diseases tend to follow a more recurved or tortuous course through the neuropil [20]. The tortuosity of a vessel segment is defined as the length along the vessel segment divided by the distance between the two end points [24]. Thus, a perfectly straight vessel segment has a value of 1.0, and the higher the number, the more tortuous the vessel. In the present study, the mean and maximum tortuosity of capillaries in each field of view was calculated and comparisons were made between the low and high OSA groups.

No significant differences were found in the mean tortuosity of vessels between the low and high OSA groups at any of the five subregions (Figure 2G). Indeed, the mean tortuosity value for all regions was close to 1.0, indicating that most vessel segments were relatively straight. Although maximum tortuosity did not differ between the low and high OSA groups (Figure 2H), the fimbria had the smallest maximum tortuosity of all five regions tested, probably because the vessel segments in the fimbria were shorter.

### 2.2. Associations Between Regular CPAP Use and Microvascular Abnormalities in CA1

The effect of regular CPAP treatment on microvascular abnormalities was investigated by comparing the non-CPAP group with the regular CPAP treatment group in the low and high OSA groups. CPAP users with high OSA had fewer string vessels than non-CPAP users; however, this difference did not reach significance (3.4 ± 0.8 vs. 6.0 ± 1.9, 95% CI [−6.46, 1.26], *t* (13) = −1.455, *p* = 0.169, unadjusted). With sample sizes of *n*_1_ = 10 and *n*_2_ = 5, a two-sided α = 0.05, and an estimated power of 80%, the estimated MDE was *d* = 1.66 and the observed effect was *d* = −0.80. As the difference between groups was not significant, the null hypothesis, that CPAP use is not associated with fewer string vessels in the CA1, was retained.

**Figure 3:**
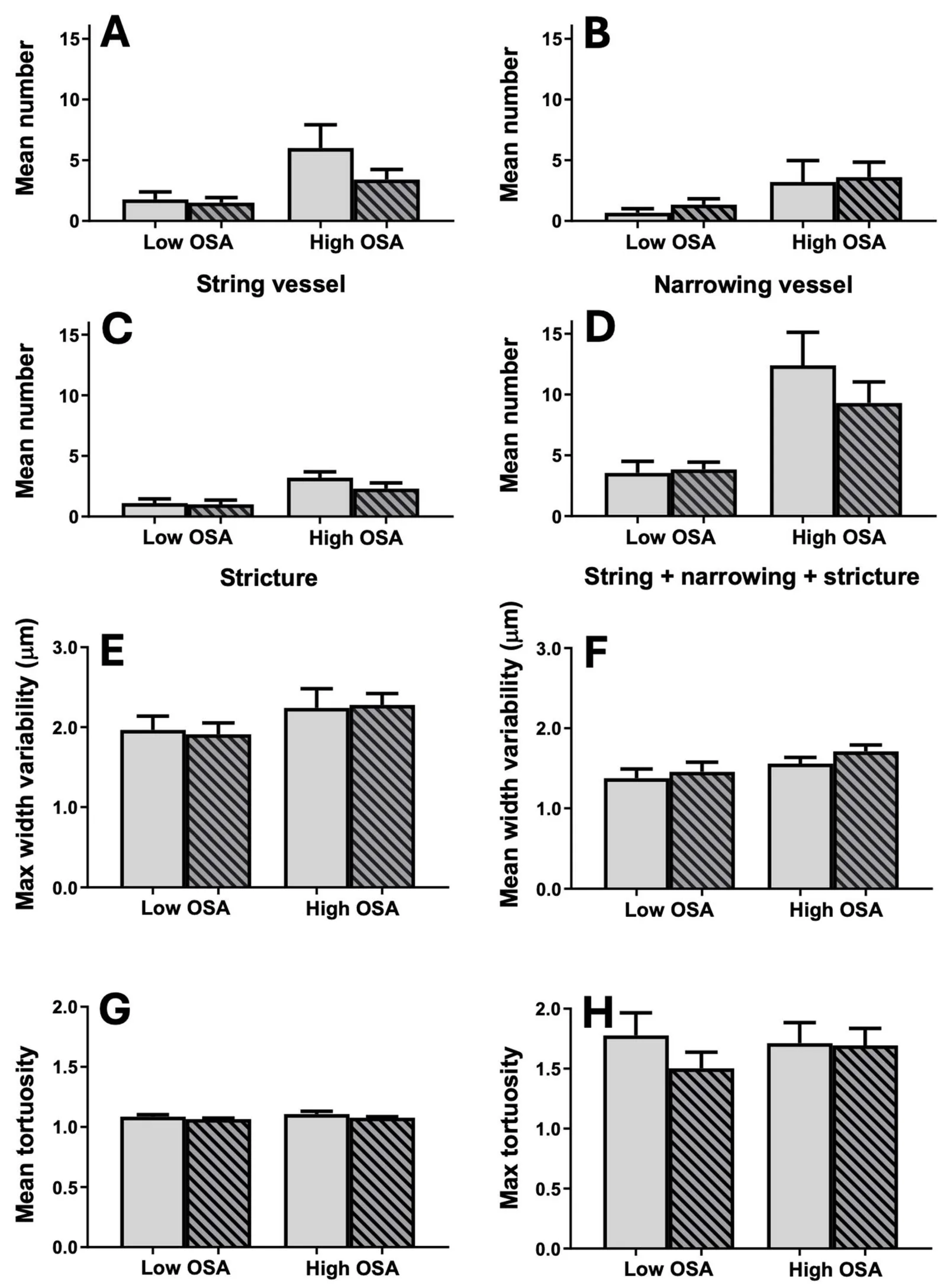
Associations between regular CPAP use and the frequency of abnormal microvessels in the CA1 in low OSA (ODI < 20 events/h sleep) and high OSA (ODI ≥ 20 events/h sleep). (**A**) String vessel counts in CA1 region between non-CPAP users and regular CPAP users in low and high OSA groups. (**B**) Narrowing vessel counts in CA1 region between non-CPAP users and regular CPAP users in low and high OSA groups. (**C**) Stricture counts in CA1 region between non-CPAP users and regular CPAP users in low and high OSA groups. (**D**) Total diseased vessel counts in CA1 region between non-CPAP users and regular CPAP users in low and high OSA groups. The width variability measures (**E**,**F**) are indicators of dilated/constricted vessels, while the tortuosity measures (**G**,**H**) are indicators of tortuous vessels. Plain bars = non-CPAP users; striped bars = CPAP users.

Indeed, no significant differences were found between regular CPAP users and non-CPAP users with respect to the frequency of string vessels (Figure 3A), narrowing vessels (Figure 3B), strictures (Figure 3C), total vessel abnormalities (Figure 3D), maximum vessel width variation (Figure 3E) or maximum vessel tortuosity (Figure 3F).

### 2.3. Associations Between Advanced Age and Microvascular Abnormalities

**Figure 4:**
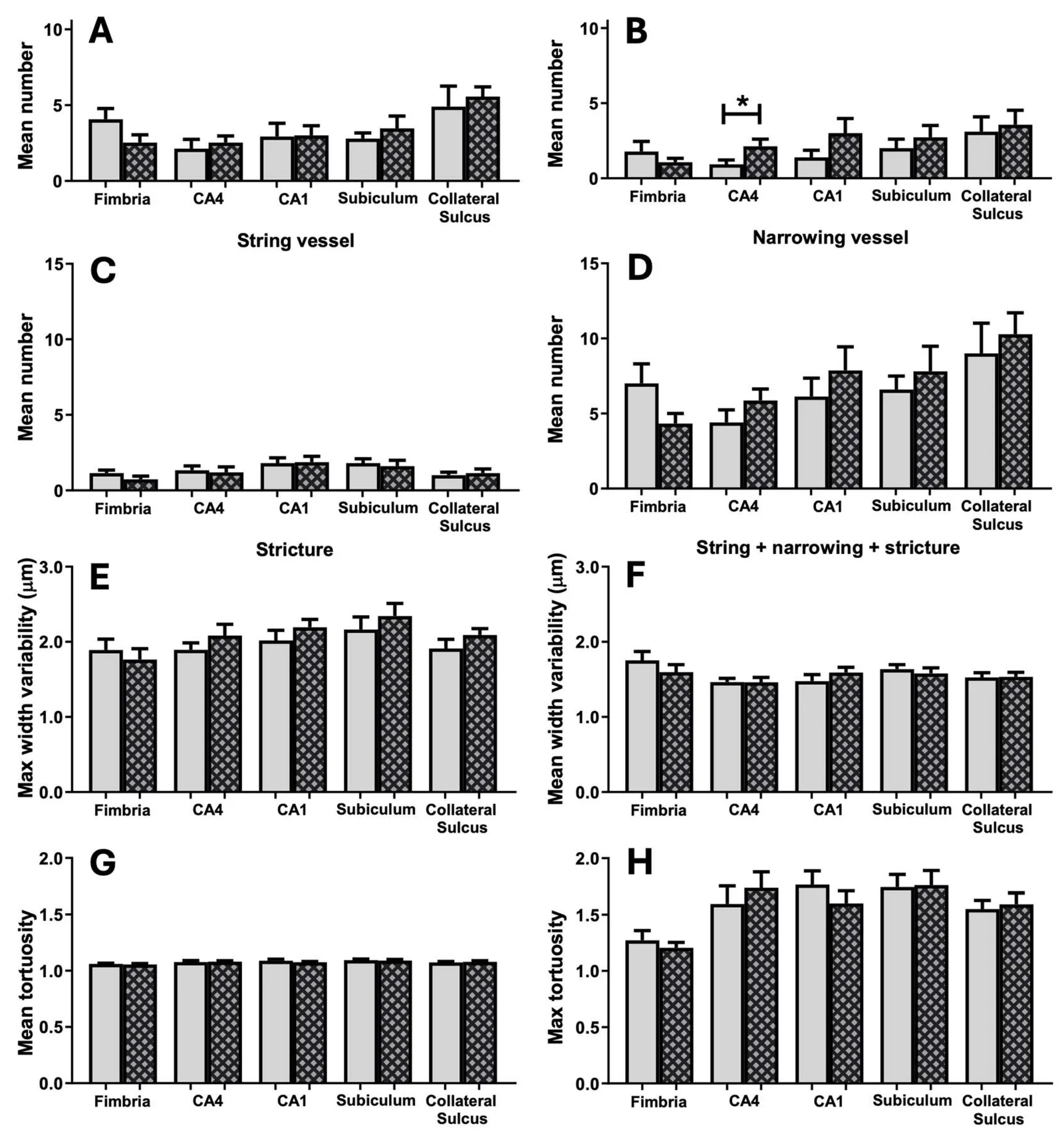
Associations between age and the frequency of abnormal microvessels in the hippocampus. (**A**) String vessel counts in younger and older aged groups across five hippocampal regions. (**B**) Narrowing vessel counts in younger and older aged groups across five hippocampal regions. (**C**) Stricture counts in younger and older aged groups across five hippocampal regions. (**D**) Total diseased vessel counts in younger and older aged groups across five hippocampal regions. The width variability measures (**E**,**F**) are indicators of dilated/constricted vessels, while the tortuosity measures (**G**,**H**) are indicators of tortuous vessels. Plain bars = low age (<67.5 years); hatched bars = high age (>67.5 years). *p* ≤ 0.05 *.

The effect of advanced age on the frequency of abnormal microvessels was analyzed by comparing tissues from patients aged < 67.5 years with those from patients aged > 67.5 years. There were 2.29-fold more narrowing vessels in the CA4 region in persons older than 67.5 years (2.1 ± 0.5 vs. 0.9 ± 0.3, 95% CI [0.05, 2.35], *t* (22.8) = 2.162, *p* = 0.041, unadjusted; Figure 4B). With 15 participants per group, a two-sided α = 0.05, and an estimated power of 80%, the estimated MDE was *d* = 1.06 and the observed effect size was *d* = 0.79. The difference between groups is consistent with the conclusion that the older group had more narrowing vessels in the CA4 than the younger group.

There were also twice as many narrowing vessels in the CA1 in the older group (3.0 ± 1.0 vs. 1.4 ± 0.5, 95% CI [−0.66, 3.86], *t* (20.0) = 1.474, *p* = 0.156, unadjusted). With 15 participants per group, a two-sided α = 0.05, and an estimated power of 80%, the estimated MDE was *d* = 1.06 and the observed effect size was *d* = 0.54. As the difference between groups was not significant, the null hypothesis that older age is not associated with more narrowing vessels in the CA1 was retained.

Apart from narrowing vessels in the CA4, age had no significant effect on the frequency of string vessels (Figure 4A), strictures (Figure 4C), total abnormalities (Figure 4D), the mean and maximum vessel width variability (Figure 4E,F), or the mean and maximum vessel tortuosity (Figure 4G,H).

### 2.4. Summary

As summarized in Table 3, the present study found that:

(i) Among the five hippocampal regions examined, the fimbria had the highest proportion of abnormal microvessels; however, their number did not differ significantly between the low and high OSA groups.
(ii) The CA1 had significantly more abnormal microvessels in the high OSA group than in the low OSA group. This difference extended to string vessels, narrowing vessels, strictures and dilated/constricted vessels. The subiculum had significantly more dilated/constricted vessels in the high OSA group.
(iii) No statistically significant differences between CPAP users and non-users were detected in this cohort.
(iv) The CA4 was the only region to show an increase in microvascular abnormalities (narrowing vessels) with advancing age.

## 3. Discussion

Ischemia is a primary cause of microvascular disease, particularly in the hippocampus, which is unusually vulnerable to hypoperfusion and hypoxia [25,26]. Since intermittent hypoxia is a defining feature of OSA, the present study examined autopsy tissue from 30 patients with clinically confirmed OSA. We found that abnormal microvessels are common in the hippocampus, and that the CA1 in patients with moderate–severe OSA exhibited more microvascular abnormalities than in patients with mild–moderate OSA. However, neither patient age nor regular CPAP use correlated with the incidence of abnormal microvessels in any subregion of the hippocampus, except for increased age in the CA4. Our results provide a new line of evidence supporting the selective vulnerability of the CA1 to ischemia. These findings have implications for the permanence of memory impairments in OSA, and for the interrelationship between OSA and AD. The implications of the present findings are discussed below.

Studies have reported that as brain tissue shrinks with age or in neurological diseases, the inelastic arterioles are forced to follow a recurved, tortuous course through the neuropil [20]. In the present study, two measures of tortuosity were employed, and the mean tortuosity was found to be equally represented in all subregions of the hippocampus, in both the low and high OSA groups, suggesting that tortuosity does not increase with OSA severity. Since the present study only analyzed capillaries, it remains possible that arterioles in the hippocampus become more tortuous with age.

Diseased microvessels, with a series of focal constrictions and dilations along their course, are found in the cerebral cortex of AD patients [16]. The present study used two metrics to quantify the degree of variability in microvessel width in OSA patients and found a trend for more variability in vessel width in the high OSA group in most hippocampal subregions, with this trend reaching significance in the CA1 and subiculum. Not only do variations in vascular diameter cause turbulence that slows blood flow [17], but also the rate of blood flow is proportional to the fourth power of the vessel radius (r^4^; Poiseuille’s Law [27]), which means that relatively minor reductions in vessel diameter can have profound effects on the rate of blood flow. For example, a dilated/constricted microvessel that narrowed from 7 µm to 5 µm would experience a 74% reduction in blood flow ([1 − 0.286]^4^ = 0.260). A single constriction is sufficient to reduce blood flow in the entire downstream segment of the vessel. In view of this, the increased variability in microvessel width in the CA1 and subiculum will increase the risk of hypoperfusion in these subregions in the high OSA group.

String vessels are the remnants of the capillary wall following the death of attendant endothelial cells due to ischemia or hypoperfusion [6,20,26]. As string vessels cannot carry blood, their presence indicates a reduced capacity to support blood flow to the immediate vicinity. In 1925, Santiago Ramon y Cajal proposed that string vessels begin from a localized stricture or narrowing of a capillary that subsequently progresses along its length to form a string [6]. The possibility that both strictures and narrowing vessels are precursors of string vessels is consistent with our observation that string vessels, strictures and narrowing vessels were much more frequent in the CA1 of the high OSA group when compared to the low OSA group. While we were unable to establish that they are precursors of string vessels, the relevant feature of strictures is that they have minimum diameters of 2.5–4.0 μm, while narrowing vessels are less than 2.5 μm, which is at the lower limit for the passage of red blood cells [28]. Consequently, strictures, narrowing vessels and string vessels should be considered together when considering reductions in the capacity to deliver blood to the hippocampus.

Several factors have been proposed to account for the selective vulnerability of the CA1 to hypoxic injury [29,30,31], including an insufficient release of neuroprotective adenosine during hypoxia [32], higher metabolic requirements of CA1 neurons, lower numbers of supportive glial cells, and higher concentrations of reactive oxygen species during ischemia [30]. These factors are presumably responsible for the increased burden of microvascular disease that we observed in the CA1 in moderate–severe OSA. We have previously shown in this Icelandic series that microvessel numbers are not increased in the high OSA group [23]. This is an important observation, as it reveals that diseased microvessels are not replaced by healthy new vessels (angiogenesis). In moderate–severe OSA, a third of the microvessels in the CA1 are so badly damaged that their narrow diameters of 2.5–1.5 μm will occlude the passage of red blood cells. These observations suggest that the ischemia associated with intermittent hypoxia may damage capillaries in the CA1 which then constrict, further limiting blood flow and worsening tissue hypoxia. Such a process may eventually result in an insufficient blood supply, leading to neuronal atrophy and death. This hypothetical scenario is consistent with our finding that the CA1 in moderate–severe OSA loses more grey matter than any other subregion of the hippocampus, with the entorhinal cortex being the next most badly affected [33]. The present study found that in addition to the CA1, the collateral sulcus displayed an increased burden of abnormal vessels in the high OSA group. Although the increase in the collateral sulcus did not reach statistical significance, the resulting proportion of abnormal microvessels is comparable to the CA1, indicating that this region, and the abutting entorhinal cortex, may also be at risk of ischemic injury.

Our earlier study [33] reported that regular CPAP use prevents tissue loss in the CA1 and entorhinal cortex in moderate–severe OSA. In view of this, we had expected to find that regular CPAP use prevents or reverses microvascular disease in these subregions. However, no significant differences were found in the burden of microvascular abnormalities between CPAP users and non-CPAP users. This apparent lack of a benefit of CPAP on microvascular disease is consistent with evidence that regular CPAP use for 6 months does not improve peripheral or central blood pressure, arterial stiffness or other haemodynamic parameters [34]. Nevertheless, we cannot exclude the possibility that if treatment adherence or the severity of OSA prior to beginning CPAP treatment had been considered, a benefit of CPAP might have been observed. Furthermore, the small sample sizes in each subgroup reduced the statistical power, making it less likely that true effects would reach significance. Thus, the observed lack of a benefit from CPAP needs to be viewed with caution.

The present study compared the microvascular morphology of patients younger than 67.5 years to those older than 67.5 years. A previous study of cerebral arterioles reported that the incidence of tortuous vessels increases after age 50 [18]. However, in the present hippocampal study, tortuous capillaries were relatively uncommon in both age groups, and we were unable to show a significant increase with age. Similarly, string vessel frequency did not increase with age in the hippocampus, which is in line with a report that string vessels in healthy normal subjects do not appear to increase with age [34]. In contrast, significantly more narrowing vessels were observed in the CA4 of patients aged older than 67.5 years. If, as we have suggested, narrowing vessels are precursors of string vessels, then the age-related increase in narrowing vessels in the CA4 may indicate the beginning of a trend towards the generation of string vessels in advanced old age. However, the proposed sequence whereby a stricture develops into a narrowing vessel which later becomes a string vessel is a working hypothesis in need of confirmation in preclinical models of ischemia.

Among the five hippocampal subregions examined, the fimbria had the highest burden of abnormality, with approximately half of the microvessels exhibiting some abnormality (Table 2). We previously found [23] that the fimbria has fewer microvessels than the other hippocampal subregions examined. The paucity of microvessels, combined with the high burden of abnormal microvessels, suggest that the axons in the fimbria, and the resident oligodendrocytes, may be particularly vulnerable to ischemic injury [35]. In the present study, however, no significant differences were found between the low and high OSA groups, or between the two age groups in the current study, suggesting that factors other than hypoxia and age are responsible for the high incidence of abnormal microvessels in the fimbria.

The hippocampus plays a pivotal role in learning and memory [36], and is selectively vulnerable to ischemic damage, with the CA1 being the most sensitive subregion [29,30,31]. In humans, the CA1 (*Cornu Ammonis* 1) is vital for the formation, consolidation and retrieval of memories, including autobiographical memories, episodic memories, visual memories, pattern separation, and spatial navigation [37,38]. Since the fimbria conveys axons from the CA1 to other limbic centres, it is also essential for memory consolidation and recall [38]. The impairments observed in OSA: autobiographical memory [39,40,41], working memory, short-term episodic memory and visual memory [40,42], are consistent with an involvement of the CA1 and fimbria. By demonstrating that abnormal microvessels are common in these two regions in high OSA, the present study may provide a neuropathological correlate of these memory impairments.

Patients with moderate–severe OSA have an increased risk of developing AD, while persons with AD are disproportionately more likely to suffer from OSA [43,44]. The present findings suggest a reason for this relationship. We previously reported that even in the absence of any observable cognitive deficits, beta-amyloid deposits are evident in the hippocampus of persons with moderate–severe OSA, and these deposits appear first at the collateral sulcus, followed by the CA1 [45]. In view of the present findings, we speculate that beta-amyloid deposits may indicate localized sites of ischemic injury caused by the hypoperfusion of abnormal microvessels. Thus moderate–severe OSA may accelerate AD pathogenesis by prematurely reducing blood flow to the CA1 and entorhinal cortex/collateral sulcus subregions, which are among the earliest sites of AD pathogenesis [46,47]. This speculation is supported by findings from AD brains and preclinical models. For example, Cullen and colleagues [48] noted that there is a close spatial relationship between capillaries and beta-amyloid deposits in AD brains and suggested that the beta-amyloid deposits might be associated with damaged microvessels. This relationship was confirmed by Richard and colleagues [49] who noted that string vessels demonstrate a remarkable co-localization with beta-amyloid deposits. Indeed, there is a strongly significant relationship between the number of string vessels in the cortical grey matter of AD brains and the number of beta-amyloid plaques [5]. Furthermore, experiments with transgenic mouse models of AD have demonstrated that chronic cerebral hypoperfusion leads to a massive increase in the number of beta-amyloid deposits in the hippocampus and cerebral cortex [50,51], whereas enhancement of cerebral perfusion reduces the number of beta-amyloid deposits by ~65% [52].

### Strengths and Limitations

The primary strength of this study is that it provides a detailed analysis of microvascular morphology in patients with clinically verified OSA and with a confirmed history of CPAP use or non-use. As far as we are aware, this Icelandic series of autopsy specimens is the only collection of its type, and therefore it provides a unique view into the histological correlates of OSA. Such detailed views cannot be obtained from current brain imaging methods (e.g., magnetic resonance imaging), as they lack the submicron spatial resolution of histological specimens. A second strength is that the present paper is part of a series of neuropathological investigations of the same hippocampal samples, enabling our observations on microvascular abnormalities to be directly related to quantitative measurements of cell layer thickness, myelination, capillary density, beta-amyloid plaques, neurofibrillary tangles and corpora amylacea [23,33,45,53]. This correspondence permits neuropathological features to be compared without the confounds usually associated with material collected from different patient groups and analyzed by different research teams.

Since this was a retrospective study of material collected for autopsy, we were limited by the availability of suitable hippocampal samples. The most significant limitation was the absence of a non-OSA control group, which prevented us from determining whether the observed microvascular abnormalities are caused by OSA or other pre-existing conditions. While a dose–response relationship exists between OSA severity and the risk of cerebral small vessel disease [54], other factors such as a history of smoking [55] and uncontrolled Type II diabetes [56] can independently alter the cerebral microcirculation. Since the clinical records available to us were incomplete, we are unable to ascertain the extent to which these other factors may have influenced the outcomes of our analyses. Consequently, an influence of concomitant vascular pathology cannot be excluded, and the observed abnormalities cannot be definitively attributed to OSA.

A second limitation relates to the sample size of 30 patients. When this group was subdivided by age, OSA severity and/or CPAP use, the smaller subgroups reduced statistical power. Consequently, the absence of a statistically significant difference does not necessarily indicate the absence of a true effect. Conversely, because multiple comparisons were performed without adjustment for family-wise error rate, the possibility of false-positive findings cannot be excluded. These results should therefore be interpreted as exploratory and hypothesis-generating rather than confirmatory. In addition, the temporal sequence we have proposed of strictures becoming narrowing vessels and then string vessels cannot be verified from this cross-sectional autopsy design and will need to be confirmed by intervention studies.

Further limitations include: (a) the sample consists entirely of Icelandic participants; (b) the CPAP usage and adherence records are incomplete; (c) the tissue samples are restricted to the hippocampus and adjacent entorhinal cortex. In view of these limitations, investigations with larger sample sizes, more complete clinical records, and a non-OSA control group are needed to confirm and extend the findings of the present study.

## 4. Materials and Methods

The materials and methods used in the present study are identical in many respects to those described in the companion paper [23]. Readers are referred to that paper for a detailed description. A summary is provided here, except where the current protocol differs from that paper, in which case those details are provided below.

### 4.1. Study Samples

The hippocampal series examined in the present study consisted of autopsy tissue from 31 clinically diagnosed OSA patients (16 males and 15 females) from Iceland who died between 1987 and 2014 [23] at an age that ranged between 41.7 and 89 years, with the average age at death being 67 years. The median oxygen desaturation index (ODI) value of 20 desaturations/h sleep was used to divide the sample into two groups (lower severity OSA group: ODI < 20 desaturations/h sleep (mean ODI 18.5 ± 3.7 events/h sleep); higher severity OSA group ≥ 20 desaturations/h sleep (mean ODI 35.9 ± 6.4 events/h sleep)). The CPAP group comprised 17 patients who had regularly used CPAP (7.4 ± 6.5 years since diagnosis), while the remainder consisted of the non-CPAP group (8.3 ± 5.6 years since diagnosis). For a description of how participants were categorized as CPAP users or non-users see [33]. Additional patient details are described in [23].

### 4.2. Double Immunohistochemistry

Formalin-fixed paraffin-embedded tissue blocks were sectioned at 20 µm, to provide more complete visualization of capillaries than the 5–10 µm thickness that is commonly used. The immunohistochemistry protocol used by our laboratory has been described elsewhere [23]. Briefly, after deparaffinization, sections were treated with antigen retrieval buffer, followed by 3 h incubation with blocking buffer at room temperature. Sections were incubated with solutions of mixed primary antibodies at their optimal concentrations (Collagen IV at 1 in 20,000, Abcam ab6586, Cambridge, UK; GluT-1 at 1 in 2000, Abcam ab15309, Cambridge, UK) for 16.5 h overnight at room temperature, followed by 3 h incubation with secondary antibody. A tertiary incubation with streptavidin-biotinylated horseradish peroxidase is followed by incubation in a solution of diaminobenzidine and hydrogen peroxide to visualize the chain of antibodies bound to the tissue. Immunolabelled sections are then washed, dehydrated and coverslipped with Depex (Electron Microscopy Sciences (EMS), Pennsylvania, USA).

### 4.3. Image Processing

The slides were scanned with the Olympus Virtual Microscopy Slide Scanning System (VS120) (microscope VS-BX; camera: VC50, Olympus Corporation, Tokyo, Japan) and Olympus VS-ASW-2.9 software (Build 13741; Hewlett-Packard Company, Palo Alto, CA, USA) at a final magnification of 10× (objective lens 20×, camera adapter mag 0.5×). Micrographs were captured from the full view images with the ‘Crop’ tool, at a resolution of 1600 × 1200 pixels, at the same five hippocampal regions (fimbria, CA4, CA1, subiculum and collateral sulcus) as described [23].

### 4.4. Definition of Abnormal Microvessels

The five types of abnormal microvessels quantified in the present study are defined and illustrated in Table 4. Three of these types have been described by others: string vessel, dilated/constricted vessel and tortuous vessel, whereas two additional types are defined in the present study: narrowing vessel and stricture. We presume that these two new types are diseased, since their irregular profile differs from the smooth profile of healthy microvessels, and their narrowed diameters will impede blood flow.

Normal microvessels have fluent walls with a consistent diameter that can range from 4 to 10 µm and with bifurcations at intervals to form two vessels. A ‘string vessel’ is a thread-like vessel segment linking two normal vessel segments/or an independent small vessel segment, with an evenly distributed narrow diameter that is less than 1.5 µm. A ‘narrowing vessel’ is a normal vessel segment that gradually tapers, the width at the narrowest point being greater than 1.5 µm but less than 2.5 µm. In a ‘stricture’ the vessel segment appears normal except for a brief constriction of 2.5–4.0 µm. A ‘dilated/constricted vessel’ exhibits considerable variability in diameter along its length, and for quantitative purposes was defined as having variability in width along its length that exceeds twice the standard deviation (the half-width of a 95% confidence interval) of all tested diameters. A ‘tortuous vessel’ follows a sinuous or recurved course through the tissue. The extent of tortuosity of a vessel segment is calculated as the length of the segment divided by the distance between the two segment end points [24].

### 4.5. Analysis of Microvascular Morphology

The width of stained blood vessels was measured manually using the Arbitrary Line measurement tool in Olympus cellSens imaging software (cellSens Dimension 1.8.1 (Build 10891), Olympus Corporation, Tokyo, Japan). For each vessel segment, the measurement was performed at its narrowest point by drawing a line perpendicular to the longitudinal axis of the vessel, extending from one vessel boundary to the opposite boundary. The linear distance was recorded as the vessel width in µm. Based on the measured vessel width, vessels were classified into four categories: string vessels (<1.5 µm), narrowing vessels (1.5–2.5 µm), stricture vessels (2.5–4.0 µm), and normal vessels (≥4.0 µm). Normal vessels were not included in the vascular abnormality analysis. The total vascular abnormality was calculated as the sum of the number of string, narrowing, and stricture vessels.

**Figure 5:**
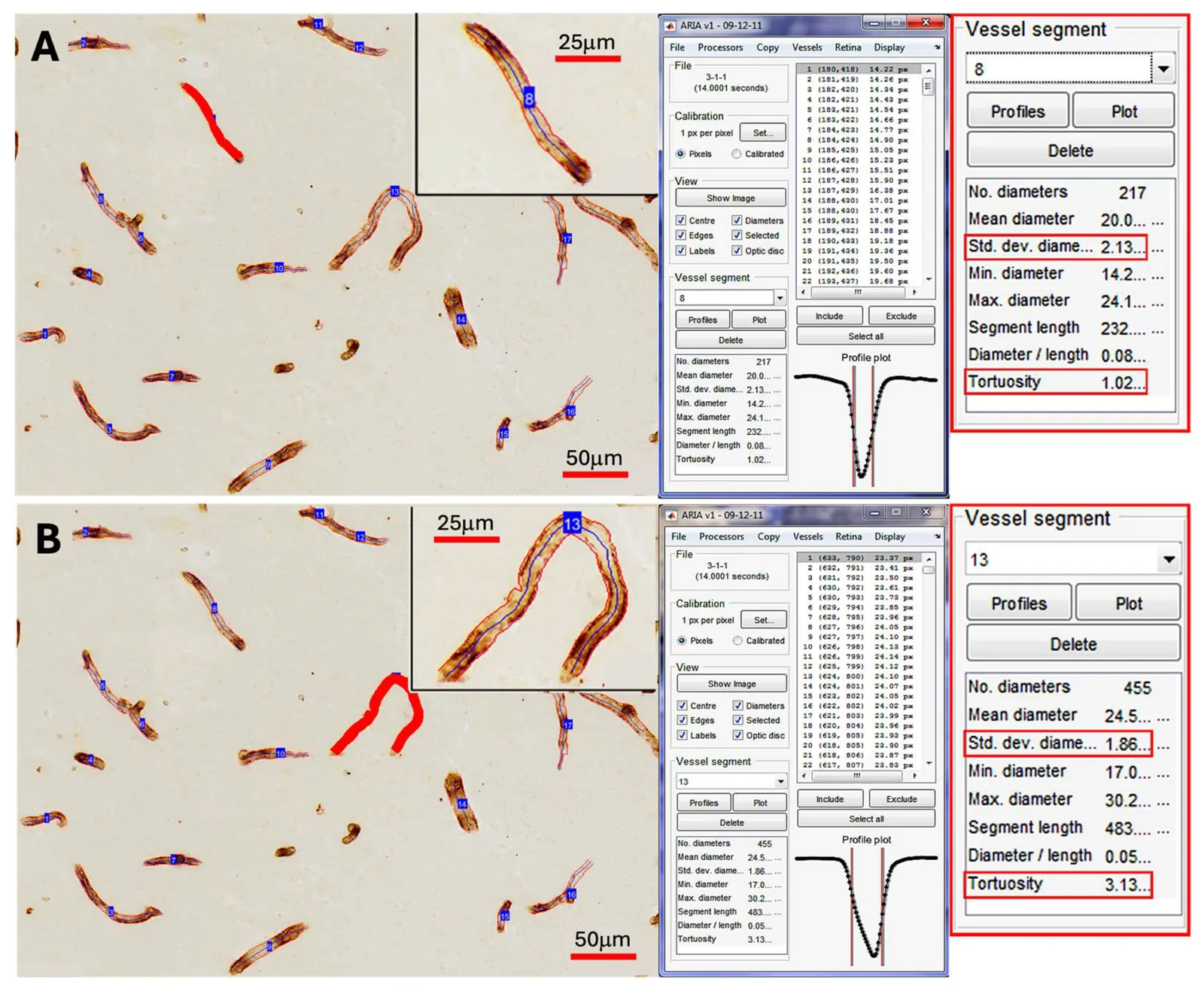
Examples of two different vessel profiles analyzed by ARIA, with the corresponding ARIA output shown on the right-hand side. A total of 19 vessel segments were identified in this micrograph, with the 8th segment (diameter measured 217 times, with mean vessel diameter variability 2SD = 2 × 2.13 = 4.26) being highlighted in (**A**) and the 13th segment (diameter measured 455 times, with mean vessel diameter variability 2SD = 2 × 1.86 = 3.72) being highlighted in (**B**). Being U-shaped, the 13th segment has a higher tortuosity than the 8th segment (3.13 vs. 1.02). The flow rate in the 13th segment will be reduced to approximately 32% (1/3.13 = 0.319) of that in a straight vessel of the same diameter.

The vessel width variation in dilated/constricted vessels and vessel tortuosity were analyzed using ARIA: Automated Retinal Image Analyzer (V1-09-12-11), an algorithm for the automated detection and measurement of blood vessels [57], run from within MATLAB^TM^ (R2017b, 9.3) with its associated Image Processing Toolbox. ARIA automatically recognizes and generates a centreline and two edge lines along each vessel segment. Diameters for each vessel segment were tested perpendicularly along the centreline every 1 pixel, as shown in Figure 5A,B; the standard deviation of diameter measurements from selected vessel segments was used to calculate the width variation of vessels that had noticeable dilations and constrictions. Segment length is the distance along the centreline, while the tortuosity is the segment length divided by the Euclidean distance between the vessel segment end points. To allow for a minimum length of vessel segment to curve, vessels with a centreline of less than 50 pixels were excluded. Data were then exported into .csv files via a separate routine.

Profiles selected by cellSens and ARIA were visually inspected to ensure that all labelled vessels within the nominated size range had been included and that the abnormal vessels had been correctly categorized. Where vessel segments had not been accurately identified or segmented, the relevant parameters were iteratively adjusted until vessel segments were appropriately detected. Once the optimal parameter settings had been established, the finalized settings were applied across all images to ensure standardized and reproducible analysis. Where necessary, inappropriately selected vessel segments were manually removed to ensure that all selected vessel segments were accurate.

### 4.6. Statistical Analysis

Statistical analysis was performed with the IBM Statistical Package for the Social Sciences (SPSS version 21). Independent samples two-tailed t-tests were performed to compare the measured parameters between the low and high OSA groups, non-CPAP users and regular CPAP users, as well as in patients grouped by age < 67.5 years and age > 67.5 years. Where the assumption of equal variances was not met, Welch’s *t*-test was used. Results are reported as mean ± standard error of means (SEM), together with its corresponding 95% confidence interval (CI), *t*-statistic, degrees of freedom, and *p*-value (unadjusted). Unless otherwise specified, the reported 95% CIs refer to the difference between group means rather than the effect size.

Effect sizes were reported as Cohen’s *d*, calculated as the difference between group means divided by the pooled standard deviation: *d* = (M_1_ − M_2_)/SD pooled, with the pooled standard deviation being calculated from the standard deviations and sample sizes of the two groups. The minimum detectable effect size (MDE) was calculated by assuming 80% statistical power and a two-sided significance level of α = 0.05. Post hoc power analyses were based on the group size (*n*), group means and standard deviations (SD), the observed effect size (Cohen’s *d*) at a significance level of α = 0.05. Statistical significance was defined as *p* < 0.05 and is indicated in the graphs by asterisks (*). Graphs were generated using GraphPad Prism 7, and data are presented as mean ± SEM.

Given the exploratory nature of this study and the hypothesis-generating objectives, no formal adjustment for multiple comparisons was applied.

## 5. Conclusions

We conclude that: (i) the CA1, and to a lesser extent the collateral sulcus, exhibit substantial increases in the proportion of abnormal (presumably diseased) microvessels in moderate–severe OSA; (ii) an increased proportion of diseased microvessels will increase the risk of localized ischemic injury; (iii) regular CPAP use did not significantly reduce the proportion of abnormal vessels; (iv) we speculate that the beta-amyloid deposition observed in moderate–severe OSA, and possibly in AD, is associated with localized ischemia resulting from inadequate blood flow in diseased microvessels.

## Figures and Tables

**Table 1:** Total number of vessel segments examined, stratified by OSA severity and hippocampal region.

	Low OSA (*n* = 15)	High OSA (*n* = 15)	Total
Fimbria	167	128	295
CA4	363	415	778
CA1	392	442	834
Subiculum	392	485	877
Collateral Sulcus	344	385	729
Total	1658	1855	3513

**Table 2:** Percentages of total vessels with an abnormal morphology, stratified by OSA severity and hippocampal subregion.

	String Vessels		Narrowing Vessels		Strictures		Total Abnormalities	
	Low	High	Low	High	Low	High	Low	High
Fimbria	36.5	26.6	13.2	14.8	7.8	10.9	57.5	52.3
CA4	10.5	7.7	5.2	6.5	4.1	5.5	19.8	19.8
CA1	6.4	14.5	3.6	11.8	4.1	8.8	14.0	35.1
Subiculum	11.7	9.9	7.9	8.2	5.6	6.0	25.3	24.1
Collateral sulcus	15.1	19.5	9.0	13.0	2.6	4.4	26.7	36.9

**Table 3:** The frequency of microvascular abnormalities according to hippocampal region, OSA severity, CPAP use and increased age.

Parameter	Region	High OSA	CPAP Use	Older Age
String vessel	CA1	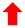	—	—
Narrowing vessel	CA1	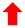	—	—
	CA4	—	—	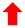
Stricture	CA1	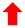	—	—
String + narrowing + strictures	CA1	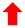	—	—
Dilated/constricted vessel	CA1	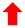	—	—
	Subiculum	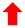	—	—
Tortuous vessel	—	—	—	—

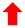
 = Frequency is increased; — = No significant change.

**Table 4:** Types of abnormal microvessels and their key morphological features as defined in the present study.

Description	Diagram	Annotation
**Normal vessel:** Fluent vessel walls with relatively constant widths of 4–10 µm.	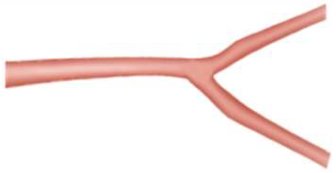	
**String vessel:** Thread-like segment linking two normal vessel segments; or an independent small vessel segment. Diameter constant, typically less than 1.5 µm.	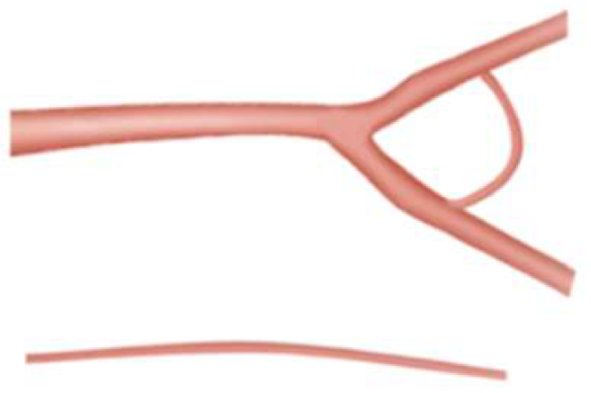	Manual linear measurements
**Narrowing vessel:** Vessel segments narrow gradually, the width at the narrowest point is between 1.5 and –2.5 µm.	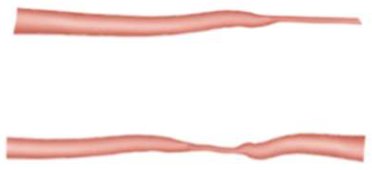	Manual linear measurements
**Stricture:** Vessel width narrows abruptly, with the width decreasing to between 2.5 µm and 4 µm.	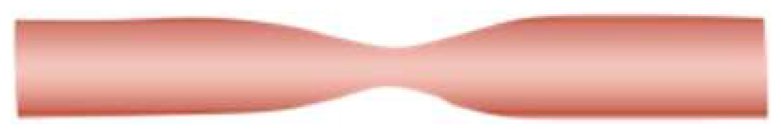	Manual linear measurements
**Dilated/constricted vessel:** Vessel has multiple dilations and constrictions. Width is measured perpendicular to the centre line (the green line) at one-pixel intervals. The variability in width of individual vessels is defined as the 95% confidence interval (2SD) for all perpendicular measures along the vessel length, in µm. Width variability was determined for every vessel in a field of view. This allowed identification of the vessel with the greatest degree of variability (maximum width variability) as well as mean width variability of all vessels in a field of view.	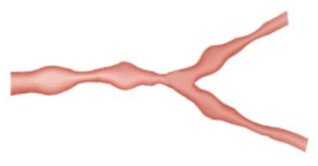	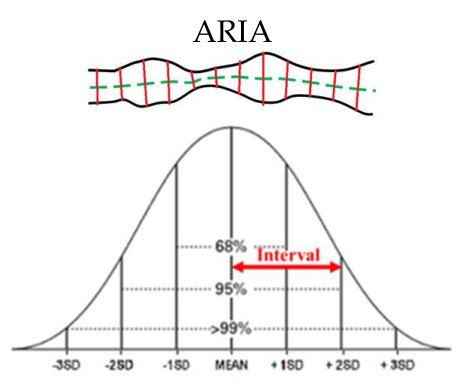
**Tortuous vessel:** The tortuosity of a vessel segment is the Euclidean distance from A to B (the green line) divided by the Euclidean distance between point A and B (the red line). A ratio of 1.0 indicates a straight vessel segment, and higher ratios indicate a less direct course. The mean tortuosity of a field of view is the average tortuosity of all selected vessel segments. The maximum tortuosity is represented by the most tortuous vessel segment in the field of view.	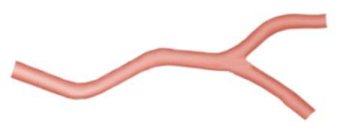	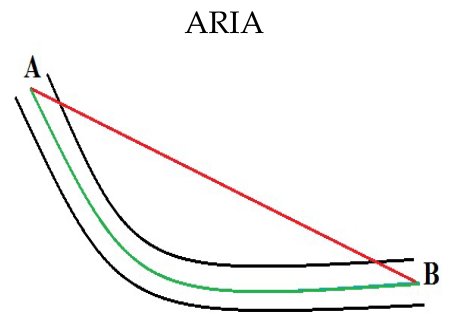

## Data Availability

The original contributions presented in this study are included in the article. Further inquiries can be directed to the corresponding author.

## Abbreviations

The following abbreviations are used in this manuscript:

AHI	Apnoea-hypopnoea index
ARIA	Automated Retinal Image Analyzer
CA1	Cornu Ammonis 1
CA4	Cornu Ammonis 4
CPAP	Continuous positive airway pressure
IH	Intermittent hypoxia
ODI	Oxygen desaturation index
OSA	Obstructive sleep apnoea
SEM	Standard error of the mean

## References

[1] Dempsey J.A., Veasey S.C., Morgan B.J., O’Donnell C.P. (2010). Pathophysiology of sleep apnea. Physiol. Rev..

[2] Sforza E., Roche F. (2016). Chronic intermittent hypoxia and obstructive sleep apnea: An experimental and clinical approach. Hypoxia.

[3] Garvey J.F., Taylor C.T., McNicholas W.T. (2009). Cardiovascular disease in obstructive sleep apnoea syndrome: The role of intermittent hypoxia and inflammation. Eur. Respir. J..

[4] Peng Y.J., Overholt J.L., Kline D., Kumar G.K., Prabhakar N.R. (2003). Induction of sensory long-term facilitation in the carotid body by intermittent hypoxia: Implications for recurrent apneas. Proc. Natl. Acad. Sci. USA.

[5] Hunter J.M., Kwan J., Malek-Ahmadi M., Maarouf C.L., Kokjohn T.A., Belden C., Sabbagh M.N., Beach T.G., Roher A.E. (2012). Morphological and pathological evolution of the brain microcirculation in aging and Alzheimer’s disease. PLoS ONE.

[6] Brown W.R. (2010). A review of string vessels or collapsed, empty basement membrane tubes. J. Alzheimer’s Dis..

[7] Challa V.R., Thore C.R., Moody D.M., Anstrom J.A., Brown W.R. (2004). Increase of white matter string vessels in Alzheimer’s disease. J. Alzheimer’s Dis..

[8] Matteis M., Silvestrini M., Troisi E., Bragoni M., Vernieri F., Caltagirone C. (1998). Cerebral hemodynamic patterns during stimuli tasks in multi-infarct and Alzheimer types of dementia. Acta Neurol. Scand..

[9] Ries F., Horn R., Hillekamp J., Honisch C., Konig M., Solymosi L. (1993). Differentiation of multi-infarct and Alzheimer dementia by intracranial hemodynamic parameters. Stroke.

[10] Ruitenberg A., den Heijer T., Bakker S.L., van Swieten J.C., Koudstaal P.J., Hofman A., Breteler M.M. (2005). Cerebral hypoperfusion and clinical onset of dementia: The Rotterdam Study. Ann. Neurol..

[11] Innes C.R., Kelly P.T., Hlavac M., Melzer T.R., Jones R.D. (2015). Decreased Regional Cerebral Perfusion in Moderate-Severe Obstructive Sleep Apnoea during Wakefulness. Sleep.

[12] Joo E.Y., Tae W.S., Han S.J., Cho J.W., Hong S.B. (2007). Reduced cerebral blood flow during wakefulness in obstructive sleep apnea-hypopnea syndrome. Sleep.

[13] L’Heureux F., Baril A.A., Gagnon K., Soucy J.P., Lafond C., Montplaisir J., Gosselin N. (2021). Longitudinal changes in regional cerebral blood flow in late middle-aged and older adults with treated and untreated obstructive sleep apnea. Hum. Brain Mapp..

[14] Hashimura T., Kimura T., Miyakawa T. (1991). Morphological changes of blood vessels in the brain with Alzheimer’s disease. Jpn. J. Psychiatry Neurol..

[15] Kimura T., Hashimura T., Miyakawa T. (1991). Observations of microvessels in the brain with Alzheimer’s disease by the scanning electron microscopy. Jpn. J. Psychiatry Neurol..

[16] Miyakawa T., Uehara Y., Desaki J., Kimura T., Kuramoto R. (1988). Morphological changes of microvessels in the brain with Alzheimer’s disease. Jpn. J. Psychiatry Neurol..

[17] Farkas E., Luiten P.G. (2001). Cerebral microvascular pathology in aging and Alzheimer’s disease. Prog. Neurobiol..

[18] Thore C.R., Anstrom J.A., Moody D.M., Challa V.R., Marion M.C., Brown W.R. (2007). Morphometric analysis of arteriolar tortuosity in human cerebral white matter of preterm, young, and aged subjects. J. Neuropathol. Exp. Neurol..

[19] Del Corso L., Moruzzo D., Conte B., Agelli M., Romanelli A.M., Pastine F., Protti M., Pentimone F., Baggiani G. (1998). Tortuosity, kinking, and coiling of the carotid artery: Expression of atherosclerosis or aging?. Angiology.

[20] Brown W.R., Thore C.R. (2011). Review: Cerebral microvascular pathology in ageing and neurodegeneration. Neuropathol. Appl. Neurobiol..

[21] Gelber R.P., Redline S., Ross G.W., Petrovitch H., Sonnen J.A., Zarow C., Uyehara-Lock J.H., Masaki K.H., Launer L.J., White L.R. (2015). Associations of brain lesions at autopsy with polysomnography features before death. Neurology.

[22] Capone C., Faraco G., Coleman C., Young C.N., Pickel V.M., Anrather J., Davisson R.L., Iadecola C. (2012). Endothelin 1-dependent neurovascular dysfunction in chronic intermittent hypoxia. Hypertension.

[23] Xu C., Owen J.E., Gislason T., Benediktsdóttir B., Ye J., Robinson S.R. (2025). Limited Microvascular Remodelling Occurs in the Aged Human Hippocampus in Obstructive Sleep Apnoea. Int. J. Mol. Sci..

[24] Bullitt E., Muller K.E., Jung I., Lin W., Aylward S. (2005). Analyzing attributes of vessel populations. Med. Image Anal..

[25] De Jong G.I., De Vos R.A., Steur E.N., Luiten P.G. (1997). Cerebrovascular hypoperfusion: A risk factor for Alzheimer’s disease? Animal model and postmortem human studies. Ann. N. Y. Acad. Sci..

[26] Mun J., Kang H.M., Park C. (2023). Cerebral chronic hypoperfusion in mice causes premature aging of the cerebrovasculature. Brain Res. Bull..

[27] Westerhof N., Stergiopulos N., Noble M.I.M., Westerhof B.E. (2019). Law of Poiseuille. Snapshots of Hemodynamics: An Aid for Clinical Research and Graduate Education.

[28] Diez-Silva M., Dao M., Han J., Lim C.T., Suresh S. (2010). Shape and Biomechanical Characteristics of Human Red Blood Cells in Health and Disease. MRS Bull..

[29] Bartsch T., Dohring J., Reuter S., Finke C., Rohr A., Brauer H., Deuschl G., Jansen O. (2015). Selective neuronal vulnerability of human hippocampal CA1 neurons: Lesion evolution, temporal course, and pattern of hippocampal damage in diffusion-weighted MR imaging. J. Cereb. Blood Flow Metab..

[30] Lana D., Ugolini F., Giovannini M.G. (2020). An Overview on the Differential Interplay Among Neurons-Astrocytes-Microglia in CA1 and CA3 Hippocampus in Hypoxia/Ischemia. Front. Cell. Neurosci..

[31] Schmidt-Kastner R. (2015). Genomic approach to selective vulnerability of the hippocampus in brain ischemia-hypoxia. Neuroscience.

[32] Ganesana M., Venton B.J. (2018). Early changes in transient adenosine during cerebral ischemia and reperfusion injury. PLoS ONE.

[33] Owen J.E., Benediktsdóttir B., Gislason T., Robinson S.R. (2019). Neuropathological investigation of cell layer thickness and myelination in the hippocampus of people with obstructive sleep apnea. Sleep.

[34] Challa V.R., Thore C.R., Moody D.M., Brown W.R., Anstrom J.A. (2002). A three-dimensional study of brain string vessels using celloidin sections stained with anti-collagen antibodies. J. Neurol. Sci..

[35] Pantoni L. (2010). Cerebral small vessel disease: From pathogenesis and clinical characteristics to therapeutic challenges. Lancet Neurol..

[36] Bartsch T., Wulff P. (2015). The hippocampus in aging and disease: From plasticity to vulnerability. Neuroscience.

[37] Bartsch T., Dohring J., Rohr A., Jansen O., Deuschl G. (2011). CA1 neurons in the human hippocampus are critical for autobiographical memory, mental time travel, and autonoetic consciousness. Proc. Natl. Acad. Sci. USA.

[38] Hanert A., Pedersen A., Bartsch T. (2019). Transient hippocampal CA1 lesions in humans impair pattern separation performance. Hippocampus.

[39] Alomri R.M., Kennedy G.A., Wali S.O., Ahejaili F., Robinson S.R. (2021). Differential associations of hypoxia, sleep fragmentation, and depressive symptoms with cognitive dysfunction in obstructive sleep apnea. Sleep.

[40] Cavuoto M.G., Robinson S.R., O’Donoghue F.J., Barnes M., Howard M.E., Tolson J., Stevens B., Schembri R., Rosenzweig I., Rowe C.C. (2023). Associations Between Amyloid Burden, Hypoxemia, Sleep Architecture, and Cognition in Obstructive Sleep Apnea. J. Alzheimer’s Dis..

[41] Delhikar N., Sommers L., Rayner G., Schembri R., Robinson S.R., Wilson S., Jackson M.L. (2019). Autobiographical Memory From Different Life Stages in Individuals With Obstructive Sleep Apnea. J. Int. Neuropsychol. Soc..

[42] Alomri R.M., Kennedy G.A., Wali S.O., Alhejaili F., Robinson S.R. (2021). Association between nocturnal activity of the sympathetic nervous system and cognitive dysfunction in obstructive sleep apnoea. Sci. Rep..

[43] Guay-Gagnon M., Vat S., Forget M.F., Tremblay-Gravel M., Ducharme S., Nguyen Q.D., Desmarais P. (2022). Sleep apnea and the risk of dementia: A systematic review and meta-analysis. J. Sleep Res..

[44] Kinugawa K. (2023). Obstructive sleep apnea and dementia: A role to play?. Rev. Neurol..

[45] Owen J.E., Benediktsdóttir B., Cook E., Olafsson I., Gislason T., Robinson S.R. (2021). Alzheimer’s disease neuropathology in the hippocampus and brainstem of people with obstructive sleep apnea. Sleep.

[46] Braak H., Braak E. (1991). Neuropathological stageing of Alzheimer-related changes. Acta Neuropathol..

[47] Thal D.R., Rub U., Schultz C., Sassin I., Ghebremedhin E., Del Tredici K., Braak E., Braak H. (2000). Sequence of Aβ-protein deposition in the human medial temporal lobe. J. Neuropathol. Exp. Neurol..

[48] Cullen K.M., Kocsi Z., Stone J. (2005). Pericapillary haem-rich deposits: Evidence for microhaemorrhages in aging human cerebral cortex. J. Cereb. Blood Flow Metab..

[49] Richard E., van Gool W.A., Hoozemans J.J., van Haastert E.S., Eikelenboom P., Rozemuller A.J., van de Berg W.D. (2010). Morphometric changes in the cortical microvascular network in Alzheimer’s disease. J. Alzheimer’s Dis..

[50] Shang J., Yamashita T., Tian F., Li X., Liu X., Shi X., Nakano Y., Tsunoda K., Nomura E., Sasaki R. (2019). Chronic cerebral hypoperfusion alters amyloid-β transport related proteins in the cortical blood vessels of Alzheimer’s disease model mouse. Brain Res..

[51] Wang L., Du Y., Wang K., Xu G., Luo S., He G. (2016). Chronic cerebral hypoperfusion induces memory deficits and facilitates Aβ generation in C57BL/6J mice. Exp. Neurol..

[52] Bragina O.A., Sillerud L.O., Kameneva M.V., Nemoto E.M., Bragin D.E. (2022). Haemorheologic Enhancement of Cerebral Perfusion Improves Oxygen Supply and Reduces Aβ Plaques Deposition in a Mouse Model of Alzheimer’s Disease. Oxygen Transport to Tissue XLIII.

[53] Xu C., Owen J.E., Gislason T., Benediktsdóttir B., Robinson S.R. (2021). Quantitative analysis of size and regional distribution of corpora amylacea in the hippocampal formation of obstructive sleep apnoea patients. Sci. Rep..

[54] Lee G., Dharmakulaseelan L., Muir R.T., Iskander C., Kendzerska T., Boulos M.I. (2023). Obstructive sleep apnea is associated with markers of cerebral small vessel disease in a dose-response manner: A systematic review and meta-analysis. Sleep Med. Rev..

[55] Salik Demirtas N., Loku E., Porschen Y., Schaffer J., Wu C.Y., Rummel C., Sommer N., Hadzic S., Weissmann N., Acker T. (2026). Hypoxia or tobacco-smoke exposure induce region-specific microvascular remodeling in the brain. Sci. Rep..

[56] Zhang Q., Liu J., Ji X. (2026). Diabetic cerebral microvascular disorder: From key pathologies to targeted interventions. Ann. Med..

[57] Bankhead P., Scholfield C.N., McGeown J.G., Curtis T.M. (2012). Fast retinal vessel detection and measurement using wavelets and edge location refinement. PLoS ONE.

